# AN EXAMINATION OF RELATIONAL COORDINATION AMONG PAID AND FAMILY CAREGIVERS IN THE ARIC COHORT

**DOI:** 10.1093/geroni/igae098.1696

**Published:** 2024-12-31

**Authors:** Katherine Ornstein, Jennifer Reckrey, Shoshana Ballew, Josef Coresh, Jennifer Wolff

**Affiliations:** Johns Hopkins University, Baltimore, Maryland, United States; Icahn School of Medicine at Mount Sinai, Icahn School of Medicine at Mount Sinai, New York, United States; New York University, New York, New York, United States; New York University, New York, New York, United States; Johns Hopkins University, Baltimore, Maryland, United States

## Abstract

The oldest-old (ages 85+) are disproportionately at risk for impaired function and reliant on support from caregivers. However, studies of caregiving typically fail to comprehensively enumerate and assess the capacity of both family and paid caregivers, shared care networks, and change over time. In this session we will describe the conceptualization and development of ARIC Cares, a caregiving study that will be embedded within the Atherosclerosis Risk in Communities Study (ARIC), a multisite prospective cohort of ~4,000 individuals ages 81-101 that has been characterized for >30 years. Existing studies of how caregiver factors relate to outcomes for older adults generally evaluate a limited set of measures specific to family caregivers (e.g., relationship to older adult). The role that paid caregivers play in meeting care needs in the community is consistently undervalued and less well studied. ARIC Cares will simultaneously assess family and paid caregivers and utilize the theory of relational coordination for understanding the dynamics of caregiving teams. ARIC Cares, which will focus on the end-of-life period and examine outcomes for older adults and their caregivers, will enumerate older adults’ care networks at 6-month intervals, in 4 diverse communities. Rigorous evidence regarding the caregiving experience, particularly focused on how teams of family and paid caregivers work together, could inform initiatives to improve quality of life among older adults and their caregivers. An understanding of these networks is critical as serious illness care, including complex dementia care, increasingly shifts to community settings where coordination between family and paid caregivers is vital.

